# Notwithstanding High Prevalence of Overweight and Obesity, Smoking Remains the Most Important Factor in Poor Self-rated Health and Hospital Use in an Australian Regional Community

**DOI:** 10.3934/publichealth.2017.4.402

**Published:** 2017-08-07

**Authors:** Helen Mary Haines, Opie Cynthia, David Pierce, Lisa Bourke

**Affiliations:** Department of Rural Health, Melbourne Medical School, The University of Melbourne, Graham St Shepparton, Victoria, Australia 3630

**Keywords:** smoking, preventable hospitalisation, rural Australia, cluster analysis

## Abstract

**Objective:**

To classify a rural community sample by their modifiable health behaviours and identify the prevalence of chronic conditions, poor self-rated health, obesity and hospital use.

**Method:**

Secondary analysis of a cross- sectional self-report questionnaire in the Hume region of Victoria, Australia. Cluster analysis using the two-step method was applied to responses to health behaviour items.

**Results:**

1,259 questionnaires were completed. Overall 63% were overweight or obese. Three groups were identified: ‘Healthy Lifestyle’ (63%), ‘Non Smoking, Unhealthy Lifestyle’ (25%) and ‘Smokers’ (12%). ‘Healthy lifestyle’ were older and more highly educated than the other two groups while ‘Non Smoking, Unhealthy Lifestyle’ were more likely to be obese. ‘Smokers’ had the highest rate of poor self-rated health. Prevalence of chronic conditions was similar in each group (>20%). ‘Smokers’ were twice as likely to have had two or more visits to hospital in the preceding year even after adjustment for age, gender and education.

**Conclusion:**

High rates of overweight and obesity were identified but ‘Smokers’ were at the greatest risk for poor self-rated health and hospitalisation.

**Implications for Public Health:**

Within an environment of high rates of chronic ill health and obesity, primary care clinicians and public health policy makers must maintain their vigilance in encouraging people to quit smoking.

## 1. Background

Poor access to health services, lack of continuity of care and reduced physician supply can explain substantial variations between geographic areas in overall health and hospital use [1]–[3]. These factors are prominent in rural Australia but it is not just rurality per se that determines poor outcomes. Key demographic characteristics contribute to an increased risk of chronic conditions and subsequent higher incidence of poor health and hospitalisation. These characteristics include increased age, male gender, single marital status, low socioeconomic status and ethnicity [4]. In Australian rural communities the population is older, poorer and more likely to be male than in urban communities [5]. People living in rural and remote areas report more negative health behaviours such as smoking, poor diet and inadequate physical exercise when compared with their urban counterparts [5],[6]. This is concerning as dietary risks, overweight and obesity followed by smoking are the three highest contributors to the burden of chronic disease in Australia [5].

Chronic conditions substantially decrease quality of life, contribute significantly to the cost of healthcare and constitute a major group of potentially preventable hospitalisations (PPH) [7]. The Australian National Health Performance Authority [7] states that six percent of hospitalisations in Australia were potentially preventable in the 2013–2014 period. PPH is a sign of serious but reducible health inequality [5] and is considered “an indicator of the accessibility and overall effectiveness of primary care” [8],[9]. In the Australian state of Victoria for example, there is wide geographic variation in prevalence of PPH attributable to chronic disease [10].

Chronic disease is often discussed in terms of four major disease groups: cardiovascular diseases, type two diabetes mellitus, cancers and respiratory disease including chronic obstructive pulmonary disease (COPD) [5]. The overall health of people with these major conditions responds well to positive health behaviours, such as smoking cessation, consumption of recommended amounts of fruit and vegetables, limited alcohol consumption and regular physical exercise [11]. Modifying entrenched poor health behaviours is not easy [12] and for primary care clinicians, hospital administrators and health policy makers local level data is important to ensure risks are flagged and addressed, efforts prioritised, and improvements noted [11].

In late 2014, a research team from the University of Melbourne, Department of Rural Health undertook a health and well-being survey [13] of residents in three of the 12 local government areas (LGAs) within the Hume region of Victoria, Australia- a region where prevalence of smoking, alcohol consumption and obesity are higher than state levels and healthy eating and exercise levels lower than state averages [14]. The work provided data specific to the respective LGAs to assist in understanding local health behaviours.

This study is a secondary analysis of that survey data and aimed to profile respondents by multiple health behaviours and then describe associations with chronic conditions, obesity, self-reported health and hospital usage. Such profiles are very useful in developing an understanding of where specifically to target local health promotion activities.

## 2. Materials and Methods

### 2.1. Design

Secondary analysis of a cross- sectional self-report health related questionnaire study administered in 2014 [13].

### 2.2. Setting

This study was set in rural Australia within three local government areas (Shires of Moira, Wangaratta and Greater Shepparton) in the Hume region of Victoria.

### 2.3. Recruitment

The questionnaire was distributed to 4,000 local households between September and October 2014. Potential participants were randomly selected from regional telephone directories and addressed to participants by name, asking the person in the household with the most recent birthday (aged of 16 years or older), to complete the survey and return it in the pre-paid envelope. All households were sent a reminder postcard 10 days later. Recruitment was supplemented by further distribution of the questionnaire in public areas of major towns and in busy waiting areas, such as medical clinics, pathology collection centres, community libraries, post offices and local corner stores in more rural areas. Simultaneously, an online version of the survey was distributed to known community contacts among the research team, such as health service staff, sporting and social groups.

### 2.4. Instrument

A 35 item questionnaire was specifically developed for this study with the inclusion of some questions previously put to residents of this region ten years earlier in the Crossroads Undiagnosed Disease Study [15]. The questionnaire comprised four sections: “about you”, “your health and wellbeing”, “health services in your region” and “your opinions on local social and cultural issues”. The full questionnaire is available from the corresponding author.

### 2.5. Analysis

**Figure 1.**
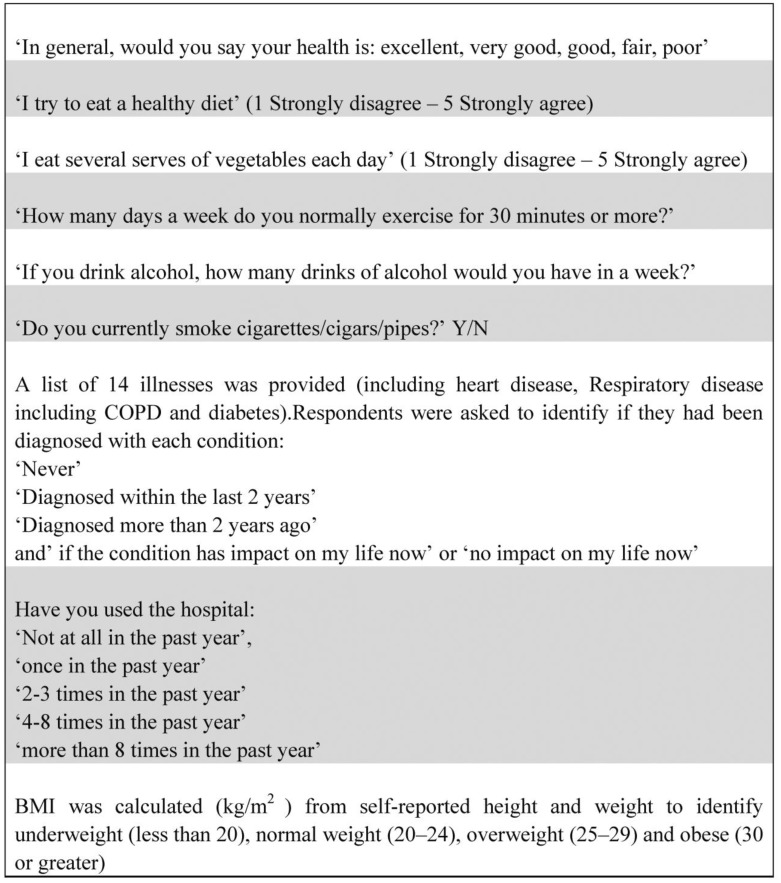
Questions relevant to this study from the community survey.

Statistical analysis was conducted using SPSS for Windows Chicago, IL, USA Version 22©. Descriptive and inferential statistics were applied to the variables of interest. The questions directly relating to the findings reported in this paper are listed in Figure 1. Given the attention was on health behaviours, three of the four key chronic conditions known to be directly influenced by health behaviours were included for analysis: heart disease, respiratory disease (including COPD) and diabetes. The fourth chronic condition, cancer was not included due to poorly reported data.

The response items “diagnosed within the last 2 years” and “diagnosed more than 2 years ago” were combined to represent “diagnosed” irrespective of whether the respondent indicated that the disease did or did not have an impact on them.

Cluster analysis using the two-step method [16], was applied to z-score transformed responses to each of the following health behaviour items in the community questionnaire:

Q21a I try to eat a healthy diet;

Q21c I eat several serves of vegetables each day;

Q16 How many days per week do you normally exercise for 30 mins or more?;

Q17 How many drinks of alcohol would you have in a week?

Q18 Do you currently smoke cigarettes/cigars/pipes?

The two step method is a powerful technique in large data sets and integrates hierarchical and partitioning clustering algorithms. This technique can detect latent relationships within the dataset between patients with multiple distinct characteristics [17]. The two step method identifies groupings or variable responses by running pre-clustering first and then hierarchical methods [18]. Schwarz's Bayesian criterion (BIC) was used to determine evidence for the model with the smaller (more negative) the BIC, the stronger the evidence [19].

Demographic characteristics, BMI, self-reported health and visits to hospital were compared between the final clusters using chi square statistics for binary outcomes and Kruskall Wallis statistics for multiple continuous outcome variables. Crude and adjusted odds ratios with 95% confidence intervals (CI) for the different outcomes were calculated using logistic regression analysis as described by Rothman [20].

### 2.6. Ethics

Approval to conduct the research was granted by the University of Melbourne Human Research Ethics Advisory Group, approval number: 1442882.2. The questionnaire was accompanied by a plain language statement which explained the voluntary nature of the research and the anonymity of the returned responses. Consent was assumed by completion and return of the questionnaire.

## 3. Results

### 3.1. Response

Of the four thousand random mail out questionnaires 1,259 were completed. The overall response rate was not calculated due to the inclusion of the responses from the supplementary recruitment methods where numbers exposed to the survey through community settings could not be calculated and subsequent response or non-response could not be accurately determined.

### 3.2. Demographics and health behaviours and chronic disease

Table 1 shows that equivalent numbers of men and women, (51% and 49% respectively) responded to the questionnaire. The median age was 60 years. Thirty percent of respondents were aged over 65 years. Consistent with the high proportion of older respondents each of the three targeted chronic conditions was reported approximately 30% respectively.

**Table 1.** Demographic characteristics of total random sample.

*n* = 1259	n	(%)
**Age** (years)		
- Median (IQR)	60 (48, 69)	
- Range	16–97	
Aged over 65	514	29
**Gender**		
- Males	682	51
- Females	655	49
**Education**		
- Year 12 completion	235	10
- Technical And Further Education certificate	135	10
- Diploma or trade	201	15
- University degree	225	17
**Health Behaviours**		
Days per week exercise for 30 minutes or more (Mean, SD)	3.5 (2.4)	
Met exercise guidelines	448	35.6
I try to eat a healthy diet	909	74.6
I eat several serves of vegetables each day	784	65.2
Number of drinks of alcohol/week (Median, IQR)	2 (0, 9)	
More than two alcoholic drinks/day	231	18.3
**Current smoker**	152	12
**BMI**		
Overweight	452	36
Obese	337	27
**Chronic Conditions**		
Heart Disease	411	32.6
Diabetes	388	30.8
Respiratory disease including COPD	380	30.2
**More than 2 visits to hospital in previous year**	271	21.5

One hundred and fifty-two people (12%) reported that they were smokers and almost 20% (n = 231) reported consuming more than two alcoholic drinks per day while almost two thirds of participants did not meet the recommended guidelines for exercise [21]. The proportion of respondents classified as overweight or obese was 63%, however the majority of respondents reported eating several servings of vegetables daily and agreement with the statement that “I try to eat a healthy diet”.

### 3.3. Hospital use

Two hundred and seventy one respondents (21.5%) used the hospital two or more times in the previous year (Table 1). Those respondents reporting having one or more of the three target chronic diseases were significantly (*p* < 0.001) more likely than those without chronic disease to use the hospital more than twice (Table 2). Similarly, being a smoker, or older aged or having a low level of education completion was significantly associated with hospital usage. However, gender and obesity were not associated with hospitalisation.

**Table 2.** Visits to hospital.

	**More than two visits to hospital in past year (n = 271)**				
	**n**	**(%)**	**X^2^**	**DF**	***P-value***
**Age Groups**					
<49	49	17.2	21.72	3	<0.001
49.01–60	52	15.6			
60.01–70	72	22.1			
>70.01	83	30			
**Gender**					
Males	161	22.1	0.43	1	0.57
Female	101	20.4			
**Education**					
Did not complete Secondary School	165	24.8	12.5	3	0.006
Completed Secondary School	26	20.2			
TAFE or Trade Cert	47	17.4			
Bachelor or higher degree	22	13.9			
**Chronic Disease**					
Heart disease/**Yes**	156	38	97.5	1	<0.001
Heart disease/**No**	115	13.6			
Respiratory (including COPD)/**Yes**	156	41.1	122.8	1	<0.001
Respiratory (including COPD)/**No**	115	13.1			
Diabetes/**Yes**	149	55	94.6	1	<0.001
Diabetes/**No**	122	45			
**Obesity**					
Yes	73	30.3	0.06	1	0.79
No	168	69.7			
**Smoker**					
Yes	47	30.9			<0.001
No	210	19.2	11.08	1	

### 3.4. Clusters

Two hundred and nineteen respondents did not answer all the required questions related to the three targeted chronic conditions and were therefore excluded, leaving a total of 1,040 respondents for cluster analysis. Using the SPSS two step cluster analysis procedure, with Schwarz's Bayesian Criterion (BIC), three clusters of people were identified according to their health behaviours with an average silhouette of 0.4. Table 3 shows the BIC changes and ratios with the three optimal clusters highlighted in bold.

Table 4 shows the centroids for health behaviours with continuous variables and proportions for categorical variables in each of the three identified groups.

**Table 3.** Schwarz's Bayesian Criterion (BIC).

Number of Clusters	Schwarz's Bayesian Criterion (BIC)	BIC Change **^a^**	Ratio of BIC Changes **^b^**	Ratio of Distance Measures **^c^**
**1**	**3,723.796**			
**2**	**2,945.733**	**−778.063**	**1.000**	**1.733**
**3**	**2,523.116**	**−422.617**	**0.543**	**1.617**
4	2,285.691	−237.425	0.305	1.580
5	2,158.388	−127.303	0.164	1.377
6	2,083.090	−75.298	0.097	1.102
7	2,020.492	−62.598	0.080	1.274
8	1,984.806	−35.687	0.046	1.402
9	1,977.277	−7.528	0.010	1.051
10	1,973.161	−4.116	0.005	1.073
11	1,973.572	0.411	−0.001	1.021
12	1,975.283	1.711	−0.002	1.066
13	1,980.734	5.450	−0.007	1.046
14	1,988.684	7.950	−0.010	1.238
15	2,007.120	18.437	−0.024	1.123
16	2,030.378	23.258	−0.030	1.063
17	2,055.948	25.569	−0.033	1.114
18	2,085.291	29.344	−0.038	1.078
19	2,117.025	31.734	−0.041	1.030
20	2,149.651	32.625	−0.042	1.042

**^a.^** The changes are from the previous number of clusters in the table.

**^b.^** The ratios of changes are relative to the change for the two cluster solution.

**^c.^** The ratios of distance measures are based on the current number of clusters against the previous number of clusters.

### 3.5. Cluster characteristics

Cluster analysis revealed three distinct groups:

**Cluster 1: Healthy Lifestyle:** Non-smokers, who eat a healthy diet, eat several serves of vegetables each day, exercise on average 4 days per week, and who average 5 drinks of alcohol per week.

**Cluster 2: Non-Smoking, less healthy lifestyle:** Non-smokers, who are less likely to eat vegetables every day, less likely to eat a healthy diet, exercise less than 3 days per week and who drink more than 8 drinks per week.

**Cluster 3: Smokers**.

**Table 5.** Cluster Characteristics (n = 1040).

	**Group 1**	**Group 2**	**Group 3**		
	**Healthy Lifestyle**	**Non Smoking, Poor Lifestyle**	**Smokers**		
	n = 654 (63%)	n = 257 (25%)	n = 129 (12%)	X^2^ (DF)	*P*-value
**Age** (Median )	59	55	53	32.5 (2) ^#^	**<0.001**
**Gender**					
Male	371 (60.4)	166 (27)	77 (12.5)	4.9 (2)	0.08
Female	282 (66.5)	90 (21.2)	52 (12.3)		
**Education level**					
Did not complete secondary school	313(48.4)	141 (57.3)	68 (52.7)	24.07 (2)	**0.001**
Completed secondary	75 (11.6)	24 (9.8)	16 (12.4)		
TAFE or Trade Cert	145 (22.4)	60 (24.4)	38 (29.5)		
Bachelor degree or higher	114 (17.6)	21 (8.5)	7 (5.4)		
**Poor Self -Rated Health**	86 (13.3)	49 (19.4)	34 (27)	16.5 (2)	**<0.001**
**Chronic Disease**					
Diabetes	174 (26.6)	62 (24.1)	30 (23.3)	1.01 (2)	0.6
Heart Disease	187 (29)	64 (25)	39 (30.2)	1.65 (2)	0.43
Respiratory Disease (including COPD)	162 (24.8)	68 (26.5)	33 (25.6)	0.28 (2)	0.86
**Obesity**	158 (29)	92 (39)	38 (31)	13.7 (2)	**0.001**
**2 or more visits to Hospital**	59 (9.6)	37 (15)	25 (20.3)	12.9 (2)	**0.002**

^#^ Kruskal Wallis.

The majority of respondents (63%) were in group one, while 25% were in group two and 12% were in group three (Table 5). There were similar numbers of men and women in each group. Several differences were identified. Firstly people in Group one: ‘Healthy lifestyle’ were older and more highly educated than people in the other two groups. There were more obese people in Group two: ‘Non-smoking, less healthy lifestyle’ and more people, in Group three: ‘Smokers’, who had two or more visits to hospital in the past year. There was a similar prevalence of each of the chronic conditions in each group.

Table 6 shows the crude and adjusted odds ratios for the three outcomes of interest between the three clusters (reference group: Healthy lifestyle).

### 3.6. BMI

When adjusted for age, gender and education those in the ‘Non-smoking, less healthy lifestyle’ (Group 2) were more likely to be classified as obese (39%), AOR = 1.9, (CI: 1.4–2.6) *p* < 0.001.

### 3.7. Self-reported health

‘Smokers’ (Group 3) were three times more likely to rate their health as poor when compared to group one, the ‘Healthy Lifestyle’ group (27%) AOR = 3.4, (CI: 2.1–5.5) *p* < 001, while the ‘Non-smoking, less healthy lifestyle’ group also reported higher odds of poor self-rated health AOR = 1.9, (CI: 1.2, 2.8) *p* = 0.03 when compared to group one.

### 3.8. Visits to Hospital

‘Smokers’ (Group 3) were twice as likely to have visited a hospital more than 2 times in the past year (20%) AOR = 2.2, (CI: 1.4, 3.5) *p* ≤ 001.

**Table 4.** Cluster health behaviours (centroids & proportions).

Cluster	Days of exercise		Number of alcoholic drinks		Tries to eat a healthy diet		Eats several serves vegetables		Smoking	
	Mean	Std. Deviation	Mean	Std. Deviation	Mean	Std. Deviation	Mean	Std. Deviation	n	%
1	3.86	2.406	4.90	6.786	4.56	0.606	4.46	0.692	0	
2	2.60	2.262	8.61	14.610	3.17	0.981	2.67	1.022	0	
3	3.36	2.515	10.62	15.891	3.64	1.088	3.40	1.338	129	100
Combined	3.49	2.442	6.53	10.838	4.10	.992	3.88	1.175	129	100

**Table 6.** Crude and Adjusted ^#^ Odds ratios for hospitalisation, self-reported health and obesity.

	Visits to hospital						Poor Self-Reported Health						Obesity					
	Crude OR	CI (95%)	*P*	Adj OR	CI (95%)	*P*	Crude OR	CI (95%)	*P*	Adj OR	CI (95%)	*P*	Crude OR	CI (95%)	*P*	Adj OR	CI (95%)	*P*
**Healthy Lifestyle**	**Ref**			**Ref**			**Ref**			**Ref**			**Ref**			**Ref**		
**Non Smoking but poor Lifestyle**	1.3	0.90–1.9	0.192	1.4	0.95–2.1	0.09	1.6	1.1–2.3	0.02	1.9	1.2–2.8	0.03	1.8	1.3–2.5	<0.001	1.9	1.4–2.6	<0.001
**Smokers**	2.1	1.3–3.3	0.001	2.2	1.4–3.5	0.001	2.5	1.6–3.8	<0.001	3.4	2.1–5.5	<0.001	1.3	0.8–2.0	0.25	1.3	0.86–2	0.2

**^#^** Adjusted for age, gender, education.

## 4. Discussion

This cross-sectional self-report study aimed to describe the modifiable health behaviours known to contribute to chronic conditions and to examine the association of those behaviours with hospitalisation, obesity and self-rated health in a rural Australian community sample. The prevalence of each chronic condition was in line with Australian population data [5]. Three clear groups were identified through a cluster analysis based on respondents' self-reported health behaviours: *‘Healthy lifestyle’*, *‘Non-smoking, less healthy lifestyle’ and ‘Smokers’* . People in the *‘Smokers’* group had the greatest risk for hospital use. In addition they were also more likely than other groups to report poor self-rated health. The *‘Non-smoking, less healthy lifestyle’* were the most likely to be obese.

Independent of the clustering and consistent with the literature [9] increasing age, low education, presence of chronic conditions and smoking were all associated with hospital use. When grouping people by these behaviours through cluster analysis, the finding that the *‘Smokers’* had twice the odds of two or more hospital visits and three times the odds of poor self -rated health when compared to the *‘Healthy lifestyle’* group is not surprising. What is surprising, is that in this rural sample, even when adjusted for age, gender and education, the *‘Smokers’* group remained at a greater risk for hospital use than the *‘Non-smoking poor lifestyle’* group who more likely to be obese than either of the other two groups. Large population data indicates that obesity increases hospitalisation with a dose effect—a pattern observed *regardless* of baseline health status, smoking status and physical activity levels [22]. Our study though, showed that in this local sample, smoking was a stronger indicator for hospital use than obesity even when adjusted for age.

With the prevalence of obesity in Australia increasing faster than any other country in the world [23] there is a justifiably strong national focus on addressing that issue [7]. Indeed in this community sample the prevalence of respondents' who were overweight or obese was 63% which is consistent with the known prevalence of 61.2% of pre-obese or obese people in the Hume region [24] and 63% in Australia [5]. Of interest was that despite two thirds of the respondents being overweight or obese, the majority agreed with the statement “*I try to eat a healthy diet*”. This finding may be reflecting recent exploratory research on food perception which showed that overweight participants tended to underestimate the caloric content of foods they perceived as healthy compared to normal weight participants [25].

Encouragingly, two thirds of respondents (63%) were clustered into the *‘Healthy lifestyle’* group. Notwithstanding, the prevalence of each of the chronic diseases and obesity in this cluster still ranged between 25–29%. Perhaps the respondents in this cluster were trying to live a healthy lifestyle in an attempt to moderate their disease. Exercise and healthy changes in diet and alcohol consumption can improve quality of life, reduce risk of recurrence or complications, and increase longevity among those with chronic disease; however with the exception of smoking cessation, the likelihood that health behaviour change in older people is maintained over the long term is low [26]. Further investigation is needed to uncover the many possible social, psychological, health care, and physical factors that may be associated with lifestyle improvement in this community.

This study shows that while the prevalence of overweight and obesity is high and of serious concern, it also remains extremely important for primary care clinicians and local policy makers not to lose sight of the negative impact smoking has on both individual health status and on the public health system. This is especially pertinent in rural Australia where Australian General Practitioner data [27] reports an increase in prevalence of smoking directly proportional to distance from large metropolitan centres. We are conscious that the prevalence of smoking reported by the respondents to our community questionnaire is an underestimation of actual smoking prevalence in the region. In this study, the overall prevalence of smoking was 12% which was lower than the known figure of 16.1% in the Hume region (in fact some local government areas included in the Hume region report prevalence of up to 27% [24]). This compares with a national percentage of 13% [5]. Even a modest reduction in line with national smoking prevalence would substantially improve health outcomes and reduce health care costs in the community where our study took place [28]. It is important therefore to continue efforts in addressing factors that reduce smoking as even in long term smokers the risk for hospitalisation can be substantially reduced within 5–14 years of quitting smoking [29].

### Strengths and limitations

There are several limitations which must be acknowledged in this study. Firstly, there are clear weaknesses in that the sample is not representative of the Hume region population. The under-representation of people in the 16 to 50 age groups is a clear bias which can be explained by the primary recruitment method of randomly selecting people via the telephone directory [30]. Younger age groups are less likely to have fixed telephone lines [31]. This is particularly important to this study as younger members of the community most likely represent the rest of the known smokers [32].

The issue of social desirability bias [33] must also be acknowledged in a study such as this. Participants are more likely to over report healthy behaviours and under report unhealthy behaviours. In particular the question relating to alcohol asked respondents “How many drinks of alcohol would you have in a week?” rather than how many “standard” drinks (10 gm alcohol) [34]. This would most likely lead to an underestimation of the amount of alcohol consumed on a weekly basis. In terms of assessing lifestyle risk factors smoking is much easier to measure than food or alcohol intake [35]. Further, the self-report nature of the questionnaire cannot guarantee the accuracy of the hospital visits to the same extent as actual matched admission data. The question put to the respondents did however differentiate visits to the emergency department and other services.

## 5. Conclusion and Recommendations

This study was able to clearly classify respondents into three identifiable groups by their modifiable health behaviours. The *‘Smokers’* group reported the greatest risk for hospitalisation and poor self-rated health. The profiles described in this study may assist local clinicians and policy makers to target health promotion efforts and may be a useful insight for other communities. Within an environment of high levels of chronic ill health and obesity and stretched resources, an important message for primary care clinicians and health policy makers is to maintain their vigilance in encouraging people to quit smoking.
